# Regulatory principles and experimental approaches to the circadian control of starch turnover

**DOI:** 10.1098/rsif.2013.0979

**Published:** 2014-02-06

**Authors:** Daniel D. Seaton, Oliver Ebenhöh, Andrew J. Millar, Alexandra Pokhilko

**Affiliations:** 1 SynthSys, University of Edinburgh, C.H. Waddington Building, Mayfield Road, Edinburgh EH9 3JD, UK; 2 Institute for Complex Systems and Mathematical Biology, University of Aberdeen, Meston Building, Aberdeen AB24 3UE, UK

**Keywords:** starch metabolism, circadian rhythms, *Arabidopsis thaliana*, mathematical modelling, systems biology, biological clocks

## Abstract

In many plants, starch is synthesized during the day and degraded during the night to avoid carbohydrate starvation in darkness. The circadian clock participates in a dynamic adjustment of starch turnover to changing environmental condition through unknown mechanisms. We used mathematical modelling to explore the possible scenarios for the control of starch turnover by the molecular components of the plant circadian clock. Several classes of plausible models were capable of describing the starch dynamics observed in a range of clock mutant plants and light conditions, including discriminating circadian protocols. Three example models of these classes are studied in detail, differing in several important ways. First, the clock components directly responsible for regulating starch degradation are different in each model. Second, the intermediate species in the pathway may play either an activating or inhibiting role on starch degradation. Third, the system may include a light-dependent interaction between the clock and downstream processes. Finally, the clock may be involved in the regulation of starch synthesis. We discuss the differences among the models’ predictions for diel starch profiles and the properties of the circadian regulators. These suggest additional experiments to elucidate the pathway structure, avoid confounding results and identify the molecular components involved.

## 1. Introduction

Starch is accumulated during the day to provide plants with a source of carbohydrates during the night, in the absence of photosynthesis [1]. In *Arabidopsis thaliana*, the diel kinetics of starch accumulation and degradation have been observed to adjust to a wide variety of environmental conditions [2–4]. In particular, it has been observed that starch levels are almost exhausted at dawn across many different light conditions. This means, for example, that starch degradation is slower in long nights (short days) than in short nights (long days) [2,3]. This diel regulation of starch breakdown allows starch to last until the end of the night, avoiding plant starvation.

It has recently been proposed that the plant regulates the turnover of starch by performing an arithmetic division calculation, setting the rate of starch breakdown during the night proportional to the quantity of starch remaining and inversely proportional to the estimated time remaining until dawn [5]. While the molecular mechanisms underlying this behaviour are unknown, the circadian clock has been shown to be an essential element in this regulation, and appears to provide the necessary timing information to the pathway [3]. This was demonstrated, for example, in experiments with various durations of the day–night cycle (i.e. different T-cycles), in which starch was exhausted approximately 24 h after dawn, irrespective of the duration of the T-cycle. Thus, starch levels at the end of the night are high in 17-h T-cycles, and starch is prematurely depleted in 28-h T-cycles [3]. Additionally, the daily starch dynamics are severely disrupted in the *lhy cca1* double mutant, which is lacking the essential clock genes *LHY* and *CCA1* [3]. Further, the premature exhaustion of starch in this mutant is accompanied by an inhibition of root extension growth at the end of the night, which can be recovered by supplying sucrose in the medium [6].

Several lines of evidence show that starch degradation rate is determined by the amount of starch remaining as well as by the clock-derived prediction of the time to dawn. For example, increasing light intensity can greatly increase starch levels at dusk, but degradation rates rise in parallel, so that starch is still almost exhausted at dawn [7,8]. Starch levels at dusk are also higher in plants grown under stable, short photoperiods than in plants grown under long photoperiods and exposed to a single, unexpected ‘early dusk’ (which creates the same, short photoperiod). The time of dusk is the same in both cases. The starch degradation rate increases in the stable, short photoperiods, such that the starch remaining at the end of the night is adjusted to the same, low level in both cases [2,3]. The metabolic pathways responsible for the synthesis and degradation of starch are well understood, and many of the involved enzymes have been identified [1,9], but the molecular mechanisms of the circadian control of the starch turnover are still largely unknown. The observed diurnal rhythms in transcripts encoding starch-degrading enzymes are insufficient to explain the regulation, because they are not accompanied by rhythmic changes in the abundance of the corresponding proteins [10,11]. Thus, it seems likely that mechanisms of post-translational regulation are responsible for coordinating starch turnover with the circadian clock.

Given the present uncertainties in the best of our knowledge regarding the clock-regulated target genes controlling starch kinetics, mathematical modelling is an attractive approach for exploring the possible general mechanisms of clock-to-starch signalling. Recently, the diurnal kinetics of starch under various photoperiods were simulated using a small phenomenological model of carbon partitioning between starch and sucrose, connected to a sinusoid-like signal representing a simple version of the clock [12]. In this model, the clock was proposed to induce large photoperiod-dependent phase shifts in the diurnal rates of starch synthesis and degradation. This simplified model is an insightful conceptual study and it can explain some minor decrease of the starch degradation in short compared with long photoperiods. However, owing to its high level of abstraction, it cannot reproduce other characteristics of starch turnover, such as the observed nearly linear degradation of starch under all photoperiods, and the suggested phase shifts with photoperiod are much larger than those observed in the transcription of starch-degrading enzymes [11] and clock components [13]. The fact that the clock input is abstracted to a sinusoidal function limits the predictive power of the model needed for the identification of the possible molecular mechanisms of the regulation of starch breakdown by the clock.

Here, we explore various possible scenarios of the regulation of starch turnover by the clock using a recently published model of the circadian clock mechanism in *A. thaliana* [14]. This creates a framework for the future analysis of potential candidate genes and mechanisms that connect the regulation of starch turnover to the circadian clock. We describe different classes of models of starch turnover and present three example models that provide a good fit to the data. The models suggest possible characteristic dynamics of the hypothesized clock-controlled regulators of starch breakdown, and predict the possible effects of mutations of clock genes on the overall kinetics of starch. Our models suggest criteria for distinguishing between different regulatory principles, and we propose specific experiments for validation of the different scenarios.

## 2. Model development

Our goals are (i) to explore the regulatory principles of circadian regulation of starch turnover, (ii) to analyse possible outcomes of the various regulatory scenarios under genetic perturbations of the circadian clock, and (iii) to suggest experiments capable of distinguishing between the various forms of regulation suggested, and of identifying the components involved in the pathway. To achieve these goals, we developed simple models of possible regulatory mechanisms controlling starch turnover, and linked these models with a detailed and experimentally validated model of the circadian clock in *A. thaliana* [14]. This approach reflects the differences in the levels of knowledge existing on the two subsystems. While the clock components are well established and are therefore represented in detail in our model, the regulatory interactions are more speculative and are correspondingly represented by simplified heuristic equations.

**Figure 1.**
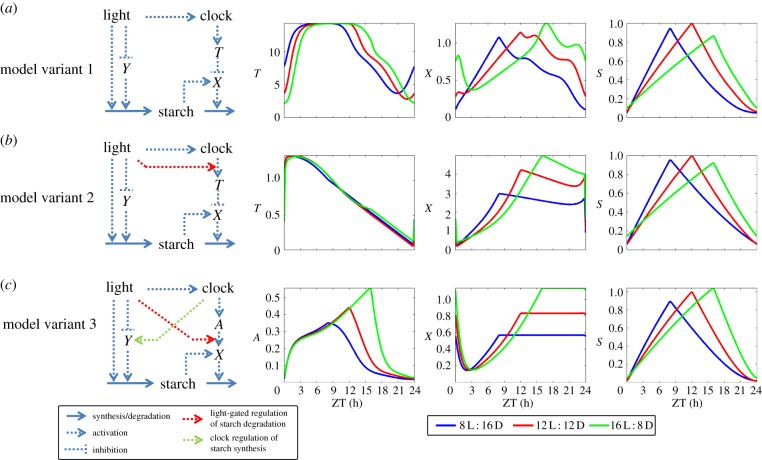
Model schematics and dynamics across photoperiods. Simplified representations of the modelled regulatory pathways are shown for the three model variants, along with the dynamics of the key components in three photoperiods. (*a*) Model variant 1, involving the continuous regulation of starch degradation by the clock through the inhibitor component *T*. (*b*) Model variant 2, involving light-gated regulation of starch degradation by the clock through the inhibitor component *T*. (*c*) Model variant 3, involving light-gated regulation of starch degradation by the clock through the activator component *A*, and clock regulation of starch synthesis through *Y*.

In the following, we take heed of the uncertainties in knowledge and assume different alternatives for key regulatory processes, each of which can reproduce the system's principle behaviours. The three model variants that we investigate in detail consist of different combinations of these alternative forms of regulation (see figure 1 for schematics).

### 2.1. Simple model of starch turnover

The observed accumulation of starch in the light and degradation during darkness suggests a simple equation describing the turnover:2.1
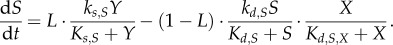


Here, *S* is the concentration of starch, *L* describes the light input by a time-dependent function assuming the values 0 (darkness) and 1 (light; see electronic supplementary material), *X* and *Y* describe the activity of components/enzymes responsible for starch degradation in the dark and synthesis in the light, respectively. In very general terms, these components represent the overall activities of enzymes and their regulators catalysing starch degradation (such as BAM, GWD, SEX4, etc.) and synthesis (e.g. APGase); *k_s,S_* and *k_d,S_* are the rate constants of starch synthesis and degradation, respectively; *K_s,S_* and *K_s,D_* are the associated Michaelis–Menten constants. The parameter *K_d,S,X_* determines the range of concentrations over which *X* is able to control starch degradation. All variables in this and following equations are dimensionless, and the time unit is 1 h. Note that starch degradation in the dark is linear in time for constant 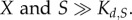 The possible mechanisms of the regulation of *X* and *Y* are discussed below.

### 2.2. Inhibition or activation of starch degradation by the clock?

Starch degradation has been observed to change in various conditions in a dynamic way, adapting to changes in photoperiod [3,11], light intensity [7,8], night temperature [4] and even unusual light conditions such as skeleton light photoperiods [3]. In all of these responses, the rate of degradation adjusts such that starch is almost but not entirely exhausted at dawn. This suggests that the underlying regulator of starch degradation, *X,* must integrate information about the time of the day (through the circadian clock) with measurements of the amount of starch remaining. In principle, two different mechanisms for conveying time of day information are conceivable: a clock-controlled ‘time-to-dawn’ signal (*T*) that is depleted 24 h after dawn, reflecting the expected time until the next dawn, or a clock-controlled component (*A*) that is accumulated during the day. High levels of *T* would inhibit starch degradation, whereas high levels of *A* would activate it (figure 1). These regulatory principles can, in a simple way, be reflected by the following equations:

The substance *T* inhibits starch degradation through decrease of *X*2.2
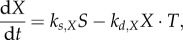
which, assuming *X* achieves a quasi-steady state for a given *S*, *T* (i.e. high *k_s,X_*, *k_d,X_*) gives2.3
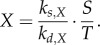
Alternatively, the day timer *A* might activate starch degradation through increase in *X*2.4
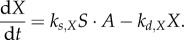


For fast dynamics of *X*, we have2.5
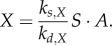
These represent two simple biochemical realizations of the arithmetic division calculation that have been proposed to underlie the regulation of starch degradation [5], and could, in principle, be replaced by alternative mechanisms that are able to perform the same calculation.

### 2.3. Continuous regulation of starch degradation by the clock?

Given that the clock can regulate *X*, either via inhibitor *T* or by activator *A*, it is then required to ask how the clock controls *T* or *A*. In this section, three possibilities for the form of this regulation are presented. The differences between the corresponding model variants are then discussed in the Results section.

One straightforward possibility is that clock components are responsible for continuously controlling the synthesis of *T* (with the same idea applying similarly to *A*)2.6
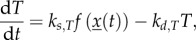
where *x*(*t*) represents the dynamics of the circadian clock components, described with the latest version of the plant clock [14], presented in the electronic supplementary material. The function *f*(*x*(*t*)) represents the effect of the clock components on *T*. For example, a model in which *T* is stimulated by the presence of the clock transcription factor LHY (and its homologue, CCA1), is described by2.7
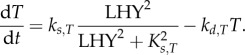
While this model is appealing in its simplicity, the strong link that this form of regulation establishes between the clock and starch degradation during the night predicts dramatic changes in starch turnover in many clock mutants. These dramatic changes are difficult to reconcile with observations (see Results). Therefore, we further consider two possible mechanisms that decouple the regulation of *X* from the clock after dusk. One way to achieve this is to introduce a weakened dependence of *T* or *A* on the clock after dusk. For example, *T* might be controlled by the clock during the day and decay during the night. For linear decay of *T* after dusk, the dynamics are given by2.8
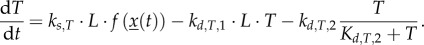


Additionally, the dynamics of *X* might be fast in the light, but slow in the dark. Restricting the adjustment of X to the light period by modifying equation (2.2) to2.9
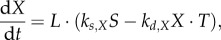
sets *X* at dusk to a value remaining unchanged throughout the night and changes in the clock output (*T* or *A*) during the night do not impact on the dynamics of starch degradation. This is an extreme case of the absence of the clock regulation of *X* during the night, which is opposite to the continuous clock signalling discussed above, and is useful to explore differences in the predicted dynamics. Of course, intermediate scenarios with reduced clock effects in the night are also plausible.

### 2.4. Clock-dependent or clock-independent regulation of starch synthesis?

Rates of starch synthesis have been observed to be reduced in longer photoperiods [2,15], suggesting regulation of *Y* with day length. The clock was shown to regulate many photoperiodic pathways (e.g. the flowering time and hypocotyl elongation pathways [16,17], and the rates of starch synthesis are modified in clock mutants [3]). Therefore, two scenarios are conceivable: a model incorporating a clock-regulated starch synthesis can be represented in most general terms by2.10
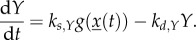


Alternatively, a clock-independent, exclusively light-regulated starch synthesis can be established through the alternation between degradation and synthesis of *Y* in the light and dark, respectively2.11
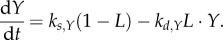


In both scenarios, a slow turnover of *Y* results in only mild changes in the levels of *Y* during one diurnal cycle, but the levels will change significantly between photoperiods, after several days of adaptation. Again, a combination of both mechanisms is plausible.

For the simulations presented in detail in Results, we considered three exemplary model variants, schematics of which are shown in figure 1. The detailed implementation is described in the electronic supplementary material. Variant 1 represents a model with continuous resetting of the starch degradation by the clock and clock-independent starch synthesis. Variant 2 decouples the clock from starch degradation after dusk by invoking light-gated regulation of *T* by the clock, according to equation (2.8), whereas variant 3 achieves similar decoupling by fixing the levels of the starch-degrading component *X* at dusk according to equation (2.9). While variant 2 incorporates inhibition of starch degradation by the clock through component *T*, variant 3 assumes activation stimulation of starch degradation through activator *A*. An additional difference is that starch synthesis is assumed to be clock-independent in variants 1 and 2 and clock-regulated in variant 3. Model parameters were chosen by fitting the models to the experimental data under various photoperiods and light conditions as presented in the electronic supplementary material. Sensitivity analysis demonstrated the robustness of the models to random variation of the parameters (see electronic supplementary material, figure S1).

## 3. Results

We present an investigation of the exemplary model variants in several stages. First, we demonstrate that all are able to reproduce the experimentally observed dynamics of starch turnover across a range of light conditions. In particular, simulated starch dynamics are compared with data from plants grown in different photoperiods [2], in skeleton photoperiods, in long and short T-cycles, and in plants confronted by an early dusk [3]. Next, we simulate a range of circadian clock mutants using the detailed model of the circadian clock, and compare these with experimental observations where available. Finally, we suggest experiments to help distinguish between the alternative regulatory principles.

### 3.1. Regulation of starch turnover in wild-type plants under various light conditions

**Figure 2.**
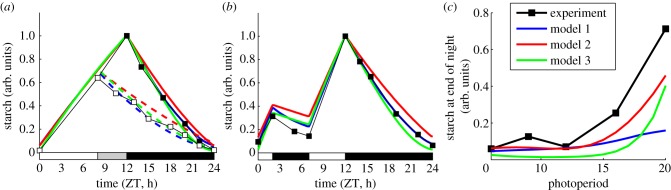
Comparison of model simulations to experimental data across multiple light conditions. (*a*) Starch turnover following an early dusk. Experimental data are taken from [3] (closed squares—12 L : 12 D, open squares—grown in 12 L : 12 D, moved to 8 L : 16 D). (*b*) Starch turnover in skeleton photoperiods. Experimental data are taken from [3]. (*c*) Starch remaining at the end of the night in different photoperiods. Experimental data are taken from [2].

In figure 2, simulated starch dynamics are compared with experimental observations (from [2,3]) across a wide range of different light conditions. All model variants are capable of describing the dynamic adjustment of starch turnover in response to early dusk (figure 2*a*). The pattern of starch turnover is also robust to the skeleton photoperiod (figure 2*b*), in which the plant is subjected to 5 h of darkness (between ZT2 and ZT7) during the day. This is a direct result of the robustness of the circadian clock in these conditions. Finally, the comparison of the models’ behaviour across multiple photoperiods (figure 2*c*) demonstrates that all three models can describe the experimentally observed increase in the starch levels at dawn in longer photoperiods. The adjustment of the dynamics of the starch-degrading agent *X* to different photoperiods is shown in figure 1, showing the expected increase that leads to faster starch degradation after longer photoperiods. In summary, all three models are able to provide a good qualitative match to the measured dynamics of starch in wild-type plants under various light conditions.

Interestingly, the levels of starch at the end of the night depend only weakly on the peak/end-of-day starch levels (see electronic supplementary material, figure S2). This is an important property shared by all three model variants, which results from the linear dependence of the starch degradation rate on the starch level (equations (2.2) and (2.4)). This agrees with the observation that the pattern of starch turnover is relatively insensitive to changes in light intensity, which influence starch synthesis rates (and hence end-of-the-day starch levels) considerably [5,7,8].

Next, we tested the behaviour of the models under various T-cycles, in which the duration of the light/dark cycle is changed while keeping the proportional duration of light and dark periods the same. All variants predict incomplete consumption of starch in 20 h T-cycles (10 L : 10 D) and the premature depletion of starch in 28 h T-cycles (14 L : 14 D; electronic supplementary material, figure S3). This agrees with experimental observations that starch turnover appears to be regulated specifically for 24-h days [3].

### 3.2. Regulation of starch turnover in clock mutants

**Figure 3.**
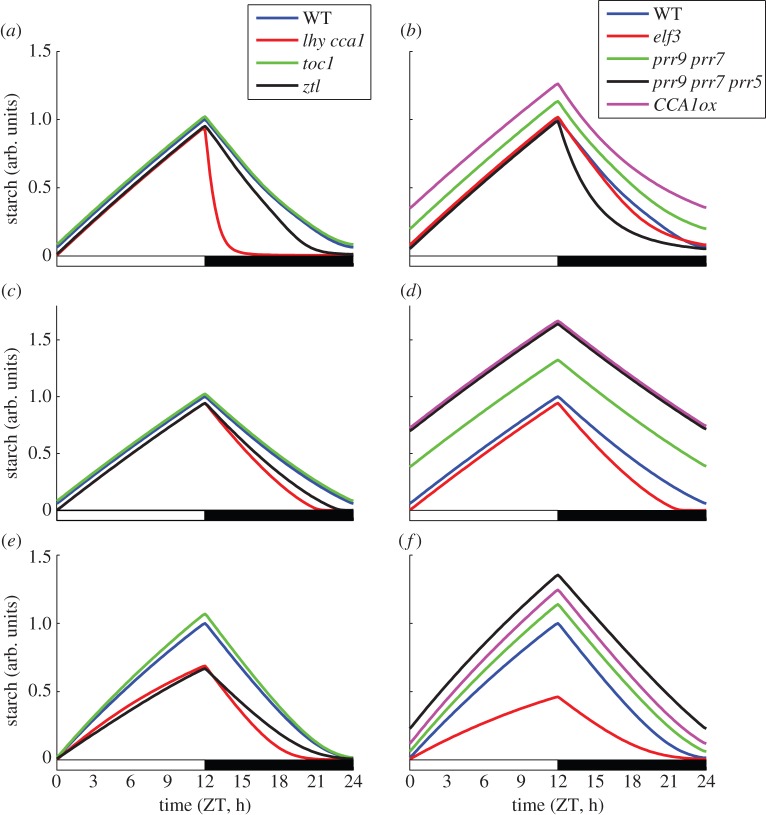
Predicted dynamics of starch turnover in clock mutants. Simulations of the clock mutants for which experimental data are available (i.e. *lhy cca1, toc1,* and *ztl*) are shown in (*a*,*c*,*e*) for model variants 1, 2 and 3, respectively. Simulations of the additional clock mutants *elf3*, *prr9 prr7, prr9 prr7 prr5* and *CCA1ox* are shown in (*b*,*d*,*f*) for model variants 1, 2 and 3, respectively.

The incorporation of a detailed model of the circadian clock allows us to investigate the effects of mutations in clock genes on the dynamics of starch turnover. Based on the parameter sets determined by a fit to wild-type data only (see above), we predict starch time-courses for a range of mutants (figure 3). The top, middle and bottom panels correspond to the predictions from model variants 1, 2 and 3, respectively. The left-hand panels display simulations of mutants for which the dynamics of starch turnover have been measured experimentally (*lhy cca1, toc1* and *ztl* [3]). The right-hand panels display simulations of a range of mutants for which only limited data are available (*elf3*, *prr9 prr7 prr5, CCA1ox* [6,18]).

The best characterized mutant is the *lhy cca1* double mutant (see electronic supplementary material, figure S4), for which two independent sets of experiments showed a starch depletion before ZT20 in L12 : D12 conditions [3,6]. All model variants predict a qualitatively similar, linear starch degradation and early depletion in this mutant. Variants 2 and 3 quantitatively match the observed time of starch depletion in this mutant, whereas in model variant 1 starch depletion occurs much earlier, with starch depleted by ZT15. This discrepancy arose from too strong an effect of the clock on starch degradation, when the simulated *lhy cca1* mutant clock model is combined with model variant 1. This particular scenario nonetheless revealed an interesting effect with more general implications (discussed below). In addition to *lhy cca1*, starch dynamics have also been measured in the clock mutants *ztl* and *toc1*. All model variants predict a marginal increase of starch levels in the *toc1* mutant and decrease in the *ztl* mutant, which exhibits high levels of TOC1 protein [19]. These predictions of the models qualitatively agree with the experimental observations [3]. The presented simulations of the models differ in some aspects of the diurnal turnover of starch, such as peak levels of starch, and the precise dynamics vary slightly upon changes in the parameter values. In summary, these results suggest that all three models provide equally good matches to the available dynamic measurements of starch turnover in clock mutants from Graf *et al*. [3].

In order to identify conditions that might allow us to discriminate between the different regulatory principles of starch turnover, we next extend our analysis to other clock mutants. Quite unexpectedly, we found that despite large differences in the mechanisms of starch regulation, the model variants give qualitatively similar predictions for the starch dynamics in a multitude of clock mutants. In particular, the mutant of both *PRR9* and *PRR7* genes, the *prr7 prr9* double mutant, has an increased level of starch, similarly to the *prr9 prr7 prr5* triple mutant (mutant of *PRR9, PRR7* and *PRR5* genes) and the CCA1-overexpressing line (*CCA1ox*; figure 3). The qualitative similarity of the predictions for various mutants can be explained by the interconnections between all clock components. For example, the *prr9 prr7, prr9 prr7 prr5* and *CCA1ox* lines all exhibit high expression levels and/or a delayed decay of *CCA1* transcript [20,21], whereas *elf3* and *ztl* have low levels of *CCA1* and *LHY* [22–24], explaining the opposite trends in the simulated starch levels in *prr9 prr7, prr9 prr7 prr5, CCA1ox* and *lhy cca1, elf3, ztl* mutants (noting the exception of *prr9 prr7 prr5* in variant 1, which results from the direct regulation of starch degradation by PRR5 in this case; figure 3 and electronic supplementary material, figure S5). This is also reflected in the opposite effects of the *lhy cca1* and *prr9 prr7* mutations on *T* and *A* in model variants 2 and 3, respectively (see electronic supplementary material, figure S6).

The extent of the effects of mutations depends on the particular parameter values, which vary among model variants. For example, under the current parameter values, the increase of *LHY* and *CCA1* levels in the *prr9 prr7 prr5* mutant has a stronger effect on the end-of-the-night starch levels in variants 1 and 2 compared with the variant 3, whereas the decrease of *LHY* and *CCA1* levels in the *lhy cca1* mutant has a stronger effect on the end-of-the-day starch levels in variant 3 than in variants 1 and 2. This can be understood by considering two differences between variants 1 and 2 compared with variant 3. First, variants 1 and 2 have a stronger clock regulation of starch degradation, such that starch levels at the end of the night in the simulated mutants differ widely from the wild-type. Second, the starch synthesis rate is clock-regulated in variant 3, whereas it is purely light-regulated in variants 1 and 2 (see Methods). Despite these differences, all model variants provide reasonable matches to existing data, both in 24 h day (figure 3) and other T-cycles (see electronic supplementary material, figure S7). Further experiments with clock mutants are necessary to discriminate between different scenarios of the clock involvement in the regulation of starch synthesis and degradation.

### 3.3. Predicted properties of the diurnal regulators of the starch turnover

The model variants involve two alternative hypothetical components responsible for the regulation of starch degradation. These are an inhibitor *T* (model variants 1 and 2), which is slowly degraded during the day, and an activator *A* (variant 3), which is accumulated during the day (figure 1). Given that both alternatives result in the models providing good agreement with multiple experimental observations, it is interesting to illustrate the predicted changes in the dynamics of these components in clock mutants with a strong phenotype, such as the *lhy cca1* mutant (with the shortest free running period) and the *prr9 prr7* mutant (with a longest free running period). Model variant 3 suggests a dramatic increase in levels of activator *A* for the *lhy cca1* mutant, whereas it decreases in the *prr9 prr7* mutant (see electronic supplementary material, figure S6*b*). Variant 2 (and, similarly, variant 1) predicts opposite changes for the profiles of the inhibitor *T* in these mutants (see electronic supplementary material, figure S6*a*), as expected given its opposite role in these model variants. These predicted profiles of the hypothetical diurnal regulators *T* and *A* may provide a basis for their identification in future experiments.

### 3.4. Predicted effects of saturation in the degradation pathway

As discussed above, there is a discrepancy between model predictions and experimental observations in the *lhy cca1* mutant for model variant 1. In particular, variant 1 predicts a rate of starch degradation that is much too high, resulting in very early depletion of starch (depleted by ZT15, compared with ZT20 in experiments). Such rapid degradation is typically observed in mutants that compromise the basic starch structure, such as the *isa1* mutant [4]. Therefore, it seems likely that model variant 1 is predicting much too high a rate of starch degradation for the *lhy cca1* mutant, which is not achievable by only altering circadian control in the plant. This suggests that the clock effect on starch degradation might normally more be restricted during the night than it is in model variant 1. Indeed, it is restricted by light-gating in model variant 2 and ‘dusk setting’ in model variant 3. Alternatively, the clock effect on starch degradation might be limited by an upper limit or saturation of the clock's effect on starch degradation rate by *X*. The simulated *lhy cca1* mutant with model variant 1 allowed us to explore this possibility. To create an upper limit to the clock's effect, we decreased parameters *K_d,S,X_* and *k_d,S_* (see electronic supplementary material). The additional adjustment of these two parameters of model variant 1 improved the fit of the simulated *lhy/cca1* mutant, delaying its starch degradation to match the data (see electronic supplementary material, figure S8*d*). The two adjusted parameters did not significantly affect model simulations under the light conditions already investigated (see electronic supplementary material, figure S8*a,b*), so the adjusted model is equally applicable in those conditions. Thus, model variant 1 can also recapitulate the starch dynamics of the *lhy/cca1* mutant, but only if the values of *K_d,S,X_* and *k_d,S_* are restricted. The adjusted model was used only in this section, to explore how saturation of the clock's effect on starch degradation would alter starch dynamics.

**Figure 4.**
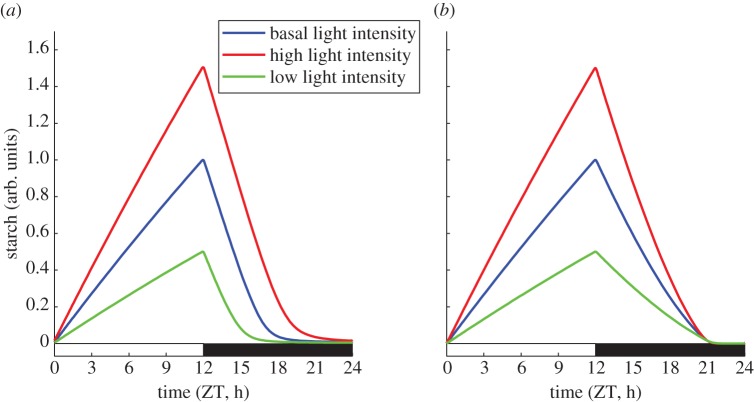
Predicted effects of the saturation in the degradation pathway. The predicted dynamics of starch turnover in the *lhy cca1* mutant at different light intensities are shown for: (*a*) model variant 1 (in which the regulatory pathway is saturated), and (*b*) model variant 2 (in which the regulatory pathway is not saturated). (Online version in colour.)

The model simulations highlighted a conceptually significant behaviour when the degradation pathway is saturated. Simulations of model variant 1 predict that under saturated conditions (e.g. in the *lhy cca1* mutant) the rate of degradation is no longer proportional to the amount of starch present, because starch degradation proceeds at its maximal rate (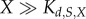), irrespective of the starch content *S*. In scenarios in which degradation is non-saturated (e.g. the wild-type), a perturbation which increases the quantity of starch at dusk elicits a compensatory change in the degradation rate that mobilizes much of the additional starch. However, in the saturated case, starch degradation will proceed at the same rate, regardless of the level of starch at dusk. A simple example of a perturbation resulting in changes in starch content at dusk is a variation of the light intensity during the day. The simulated response of the *lhy cca1* mutant to this perturbation is shown in figure 4*a* for model variant 1 (with adjusted parameters). Here, saturation of degradation occurs, and starch exhaustion occurs at different times depending on the level of starch remaining at dusk. By contrast, models in which the pathway is not saturated predict starch depletion at the same time, regardless of starch levels at dusk. This is shown in figure 4*b* for model variant 2, again in the *lhy cca1* mutant.

The results presented here use the *lhy cca1* mutant as a specific example in which the two models suggested above can be distinguished by a simple experiment: changing the light intensity. However, the distinction is general and may allow identification of other scenarios in which the clock is not the primary regulator of starch turnover. This may be relevant, for example, in mutants of the starch degradation pathway (see below), or in long photoperiods. Additionally, careful design of experiments will be needed to control for other regulators of starch metabolism, beyond those considered here, that could also bypass normal regulation. In the context of the experiment suggested above, careful design would involve growing plants at one light intensity and then shifting them to higher or lower intensity for one light period. Because starch turnover is a central component of the plant's metabolism, it is expected to be regulated by a wide range of inputs, which might lessen the impact of clock regulation. For example, it is known that modulating sugar supply to and export from leaves can affect the rate of starch degradation [1]. A switch from a clock-regulated to a non-clock-regulated mode may also explain why the introduction of a constitutively active AGPase to increase starch synthesis and starch level at dusk leads to a higher rate of starch degradation in short photoperiods and low irradiance, but not in longer photoperiods or in high light [25].

### 3.5. Distinguishing between ‘light-gated regulation’ and ‘continuous regulation’ models of circadian control of starch turnover

A key structural difference between the model variants is in the presence or absence of light-gated regulation of starch degradation. ‘Circadian gating’ refers to the rhythmic modulation of biological responses, such that the response to a fixed external stimulus has greater amplitude at a particular phase of the circadian clock [26]. Model variants 2 and 3 show the inverse: circadian regulation of *T* and *A* has altered amplitude depending on the light input. Although in model variant 1 *X* is regulated continuously by the clock (through regulation of *T*), *X* is decoupled from the clock after dusk by light-gating in model variants 2 and 3. The light-gated model variants were introduced to provide model alternatives in which the clock and starch degradation are less tightly linked than in continuous-regulation models. In principle, similar logic may apply if regulation by light is replaced by another temporal cue. In the following, we investigate different behaviours emerging from these alternative forms of regulation, focusing on the expected dynamics of starch degradation.

We begin by observing that simulated starch turnover was much more strongly perturbed in clock mutants (especially the *lhy cca1* mutant) in model variant 1 (with continuous regulation) than in variants 2 and 3 (with light-gated regulation). Intuitively, this is because the effects of clock mutations on the output dynamics during the night affect starch degradation in model variant 1, but not in variants 2 and 3 (see electronic supplementary material, figure S9). In order to demonstrate that this conclusion is a general one, we created three additional instances of model variant 1, with identical structures and parameters except for the form of the clock regulation term *f*(*x*(*t*)) in equation (2.6) for *T* (see electronic supplementary material). These models were selected on the basis of their ability to fit the basic photoperiodic behaviour discussed above (see electronic supplementary material, figure S10). As shown in the electronic supplementary material, figure S11, in all cases, these models predict that the maximal-simulated rate of starch degradation will be increased by at least approximately twofold in at least one clock mutant for continuous regulation models, whereas the increase is much less for the light-gated model variants (2 and 3). Therefore, we conclude that light-gated models are intrinsically more robust to clock mutations than continuous-regulation models.

**Figure 5.**
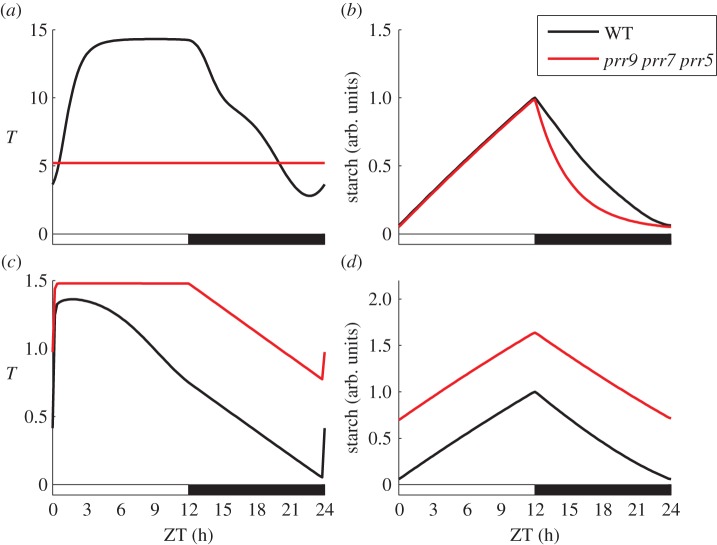
Differences between model predictions of starch dynamics in the *prr9 prr7 prr5* triple mutant. (*a*,*b*) The dynamics of *T* and starch in model variant 1. (*c*,*d*) The dynamics of *T* and starch in model variant 2. (Online version in colour.)

It is also interesting to consider differences in model predictions for cases in which the clock output to starch has been rendered arrhythmic in light–dark cycles. This might occur if the direct clock outputs are knocked out, or if the clock mechanism itself has been strongly perturbed. An example of a strong clock perturbation is the *prr9 prr7 prr5* triple mutant, in which many different clock outputs are arrhythmic [21,27]. Therefore, we can use simulations of the *prr9 prr7 prr5* triple mutant to compare the effects of an arrhythmic output on the dynamics of starch turnover in the different model variants, even when the variants use different clock outputs. The results of this comparison between model variants 1 and 2 are shown in figure 5 (variant 3 behaves similarly to variant 2; figure 3). While the difference in starch levels throughout the diurnal cycle is clear, there is also a dramatic qualitative difference in the dynamics of starch degradation. In particular, exponential degradation of starch is predicted by model variant 1, whereas a near-linear pattern of starch degradation is predicted by model variant 2. The exponential degradation of starch in model variant 1 is simply a result of the feedback from starch levels to *X* in conditions where *T* is not changing.

In summary, we have shown that light-gated models are expected to be intrinsically more robust to clock mutations than continuous-regulation models. In addition, we have identified characteristic profiles of starch degradation for both the light-gated and continuous-regulation models in cases where the clock output to this pathway is arrhythmic.

### 3.6. Starch turnover in starch-excess mutants

**Figure 6.**
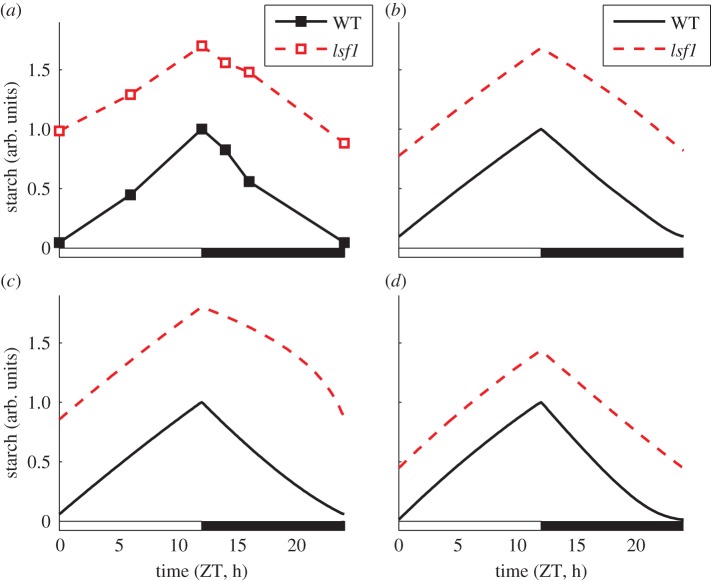
Comparison of model simulations and experimental data in the *lfs1* mutant. (*a*) Measured dynamics of starch turnover in WT and *lsf1*, taken from [28]. (*b–d*) Predicted dynamics of starch turnover under these conditions in model variants 1, 2 and 3, respectively. (Online version in colour.)

We also explored the possible effect of the mutations of the starch-degrading enzymes on the overall kinetics of starch. Figure 6 shows the increase of the starch level in a starch-excess mutant (modelled by a decrease in the starch degradation parameter *k_d,S_*) compared with experimental data of the diurnal turnover of starch in the *lsf1* mutant [28]. The simulated dynamics of starch during the night are similar to wild-type, but with elevated starch levels, consistent with the data for this, and other starch-excess mutants impaired in the functionality of enzymes involved in the starch degradation pathway, such as *sex4* [5,28,29] *isa3* [30], *bam3, bam4* [31], *dpe2* [32] and *mex1* [33].

One interesting aspect of the simulations presented in figure 6 is the increased rate of starch degradation towards the end of the night predicted by model variant 2. This is the result of dynamic adjustment of the starch-degrading component *X* after dusk in response to the excess of starch, as illustrated in the electronic supplementary material, figure S12. This cannot occur in variant 3 where *X* is fixed at dusk. Additionally, while model variant 1 also dynamically adjusts *X* in response to the excess of starch, the degradation pathway is saturated by the high starch levels, so the effect on the rate of starch degradation much less pronounced.

While this model prediction is relatively straightforward, it cannot be tested without a higher temporal resolution of measurements than is commonly available. In addition, similar to the scenario presented above for clock mutants, it is necessary to test whether the modelled regulatory pathway is active in these mutants. This can be done in a variety of ways, for example by changing the light intensity as described above to identify cases where control of the pathway is saturated. Once again, if the pathway is not active, then we predict that the rate of degradation will not adjust to different levels of starch.

## 4. Discussion

Higher plants have evolved complex regulation of transitory carbon stores on several timescales [1,34]. It is evident that this regulation depends on cues from the circadian clock as well as the ambient light conditions. However, although the molecular mechanisms of the clock are now mostly understood, it is still largely unknown how clock outputs interact with light signals to achieve metabolic regulation. To gain insights into this diurnal regulation and derive experimentally testable predictions, we pursued a systems biology approach by developing a mathematical modelling framework for describing the regulation of starch synthesis and degradation, which reflects the divergent degree of current knowledge of the involved subsystems. Using this modelling framework, we created three model variants, which we investigated in detail. All model variants were capable of describing the dynamics of starch turnover in wild-type plants in a range of conditions, including skeleton photoperiods and early dusk protocols [2,3,15]. Additionally, the models all made qualitatively similar predictions of the dynamics of starch turnover in a variety of clock mutants, which broadly matched available experimental observations [3,6,18]. In summary, our modelling framework provides a variety of sensible models of starch turnover.

Hitherto, mathematical modelling studies of plant carbon and energy metabolism were mainly focused on describing steady-state or short-term dynamic behaviour of important subsystems, including the Calvin–Benson cycle [35–37], the photosynthetic electron transport chain [38], a combination thereof [39] or aspects of the starch degradation pathway [40,41]. Recently, however, two modelling studies have investigated the regulation of starch turnover in diurnal cycles. In one study, it was proposed that starch degradation is regulated by a biochemical circuit that performs an arithmetic division calculation, resulting in the rate of starch degradation being set proportional to the quantity of starch remaining and inversely proportional to the expected time until dawn [5]. The models that we have presented here incorporate such a mechanism as a key component, and go beyond this to investigate how timing information may be transmitted to this pathway from the circadian clock. In addition to this study, a simplified model has been presented by Feugier & Satake [12], addressing the question of how energy and carbon metabolism are regulated over a longer period encompassing several days. While this highly simplified model provides valuable insights into how photoperiod-induced phase shifts may adapt starch synthesis and degradation rates to different day lengths, it does not provide a mechanistic understanding of how clock- and light-induced signals are integrated to achieve metabolic regulation. Moreover, because the phase shift was calculated for each photoperiod based on the assumption that plants minimize sucrose starvation, sudden perturbations, such as skeleton and early dusk protocols, cannot be reproduced. In contrast to this simplified approach, our modelling framework incorporates mechanistic details of the circadian clock and suggests possible regulatory links, and can thus realistically describe and predict the effect of single and multiple knockouts of core clock genes, as well as correctly simulate long- and short-term adaptation to various light protocols.

At least within the range of conditions tested experimentally, the models presented here would be suitable to include in larger-scale plant growth models. Starch pools already feature in plant models developed in crop science, and our approach could contribute to ‘next-generation’ models that balance effective dynamics with greater mechanistic detail [42,43].

Given the similarity of model predictions across a range of conditions, it was necessary to consider what additional experiments might distinguish between the model variants. The variants differed in the degree of the clock's effects on starch synthesis and degradation. The resulting, quantitative differences in the models’ predicted starch dynamics among clock mutants are testable, by extending experimental studies already reported [3,6,18]. For example, an important observation was that model variant 1 could only explain mutant data if starch degradation was assumed be saturated. A resulting model prediction is that in such cases the rate of starch degradation cannot be adapted to compensate different end-of-day starch levels. In particular, two models (variants 1 and 2) with similar behaviour in the wild-type displayed fundamentally different responses to changes in light intensity in the *lhy cca1* mutant—a prediction that can easily be tested. Beyond this particular example, experiments of this type may help us identify cases in which the normal clock regulation of starch degradation is inactive. This may be especially important in distinguishing between the direct effects of clock mutations on this pathway (through active regulation), and the indirect effects of clock mutations (through whole plant metabolism and growth). Circadian rhythms control at least 30% of *Arabidopsis* genes [44,45], so we expect that multiple clock-regulated processes in addition to starch regulation will limit vegetative plant growth when their rhythms are mis-timed [46,47].

The models’ ability to match experimental observations across a wide range of conditions suggests that they capture the essential properties of the pathway behaviour, but this in itself raises several questions about the molecular mechanisms that might realize this behaviour. The models considered the temporal regulation of starch degradation either through the inhibitor *T* or the activator *A*. The dynamics of the inhibitor *T* and activator *A* in the different model variants are very distinct, but both components perform the same role in the network. Molecular or genetic screens will now be required to identify such components. Predicting the likely behaviour of *T* and *A* in the wild-type, and the clock mutants will allow such studies to prioritize the molecular candidates for these hypothetical regulators. We expect that understanding their particular mechanisms will benefit from more detailed, dynamic models of the full metabolic pathway of carbon assimilation and sugar–starch partitioning [48].

The mechanistic basis for a feedback from starch levels is similarly unknown [1]. A central assumption in the models is that starch levels (expressed by the variable *S*) can be sensed and exert a feedback regulation on starch dynamics. This feedback is necessary in order for the observed adjustment to altered starch levels, for example under changing intensities and durations of light. However, molecular mechanisms for sensing starch content or the subsequent signalling are unknown. It is likely that the detailed molecular mechanisms of these processes are much more complicated, considering that starch is a semi-crystalline structure and only parts of the surface are ‘visible’ and accessible for a number of granule-bound starch synthesizing and degrading enzymes [49], an important detail which is not reflected in the presented models. As our knowledge of starch sensing and signalling mechanisms increase, it will become necessary to reflect the semi-crystalline nature and the geometry of starch granules. Nonetheless, the modelling framework developed here will inform the design and interpretation of future experiments, and thus aid the identification of pathway components as well as regulatory principles, as suggested for clock components [50].
